# Benefits of Point-of-Care Ultrasound for Assessing Gastric Contents

**DOI:** 10.31486/toj.26.0002

**Published:** 2026

**Authors:** Joseph Koveleskie, Sydney Labat, Matthew Palascak, David Broussard, William Sumrall, Bobby D. Nossaman

**Affiliations:** ^1^ Department of Anesthesiology and Perioperative Medicine, Ochsner Clinic Foundation, New Orleans, LA; ^2^ The University of Queensland Medical School, Ochsner Clinical School, New Orleans, LA

**Keywords:** *Fasting*, *gastric emptying*, *gastrointestinal contents*, *point-of-care systems*, *preoperative care*, *respiratory aspiration of gastric contents*, *stomach*, *ultrasonography*

## Abstract

**Background:**

Point-of-care ultrasound (POCUS) has emerged as a valuable bedside tool for assessment of gastric contents in perioperative and emergency settings. Accurate identification of gastric volume and composition is critical for estimating pulmonary aspiration risk, particularly when fasting history is unreliable or gastric emptying is impaired. Visualization of the gastric antrum enables qualitative grading and semiquantitative estimation of gastric volume, offering a practical alternative to traditional assessment methods.

**Case Report:**

An 85-year-old female with multiple comorbidities—including chronic obstructive pulmonary disease, heart failure, chronic kidney disease, and diabetes—presented with small bowel obstruction. Despite intrahospital fasting, preoperative gastric POCUS demonstrated substantial gastric contents consistent with a full stomach. Sedation was administered to facilitate nasogastric sump tube placement, resulting in effective gastric decompression. The patient underwent exploratory laparotomy with lysis of adhesions and ventral hernia repair without intraoperative complications or vasopressor requirement. Postoperatively, she was transferred to the surgical intensive care unit in stable condition. Given the patient's advanced age and cardiopulmonary comorbidities, ongoing management emphasized careful fluid balance and continued gastric decompression.

**Conclusion:**

Gastric POCUS provides high diagnostic accuracy for detecting clinically significant gastric volumes associated with aspiration risk and maintains reliability across diverse patient populations. The ability of gastric POCUS to support dynamic, real-time decision-making enhances patient safety, informs airway management strategies, and improves perioperative workflow efficiency. Future advances, including artificial intelligence–assisted interpretation, are expected to further expand the role of gastric POCUS in anesthesia and critical care practice.

## INTRODUCTION

Point-of-care ultrasound (POCUS) is a significant advancement in bedside diagnostics within contemporary medical practice, providing clinicians with an efficient, noninvasive way to visualize anatomic structures in real time.^1,2^ One notable application is the assessment of gastric contents, and gastric POCUS is developing into a vital tool in perioperative and emergency settings.^1,2^ Accurate evaluation of gastric contents is crucial for assessing the risk of pulmonary aspiration, a serious complication associated with general anesthesia and procedural sedation.^1,3^

Historically, assessment of gastric emptying relied upon patient history, physical examination, or radiographic imaging, such as x-rays. However, these approaches are frequently subjective, require considerable time, or involve exposure to ionizing radiation. By visualizing the gastric antrum with a low-frequency curvilinear probe, clinicians can reliably distinguish an empty stomach from one containing clear fluids and/or solid material.^4^ This qualitative (grade 0 to 2; Table) and semiquantitative assessment can complement fasting guidelines, particularly when fasting history is uncertain or gastric emptying may be impaired.^4-6^

**Table.** Perlas Qualitative Grading System Using Point-of-Care Ultrasound (POCUS) for Assessment of Gastric Contents and Aspiration Risk^4,5,6^

Perlas Grade	Gastric Antrum Appearance in Supine Position	Gastric Antrum Appearance in Right Lateral Decubitus Position^a^	POCUS Interpretation of Gastric Content	Aspiration Risk
0	Empty (collapsed, flat, or bull's-eye; no visible contents)	Empty, no content visible	Empty stomach or basal secretions	Low risk: standard induction safe
1	Empty or trace fluid (<1-2 mm hypoechoic layer)	Clear fluid visible, distends with gravity	Small amount of clear fluid (<1.5 mL/kg)	Low-intermediate risk: usually safe for standard induction in elective/low-risk patients
2^b^	Fluid or solid content visible already in supine position	Substantial distension^c^ with fluid ± solids	Large amount of clear fluid (>1.5 mL/kg) ± solid/food particles	High risk: treat as full stomach (rapid-sequence induction, awake intubation, or delay the case if possible)

^a^The right lateral decubitus position is the more sensitive view; fluid moves dependently into the gastric antrum.

^b^Grade 2, with content visible in both positions, is the strongest predictor of a full stomach and is almost never seen in truly fasted patients. Solids appear as mixed echogenicity with a frosted-glass pattern or air shadowing. The fluid threshold of >1.5 mL/kg ± solid/food particles for increased pulmonary aspiration risk is derived from validation studies correlating antral cross-sectional area measurements with suctioned volumes.

^c^Substantial distension is defined as an antral cross-sectional area >5.3 cm^2^ in a 70 kg patient.

This case report and literature review examine the advantages of POCUS for gastric content assessment.

## CASE REPORT

An 85-year-old female with history of chronic obstructive pulmonary disease (on 2 L home oxygen therapy), heart failure, hypertension, chronic kidney disease, diabetes, and hyperlipidemia presented to the emergency department with small bowel obstruction complicated by hiatal hernia. Initial conservative management with bowel rest and intravenous fluid hydration failed, prompting surgical intervention.

**Figure 1.**
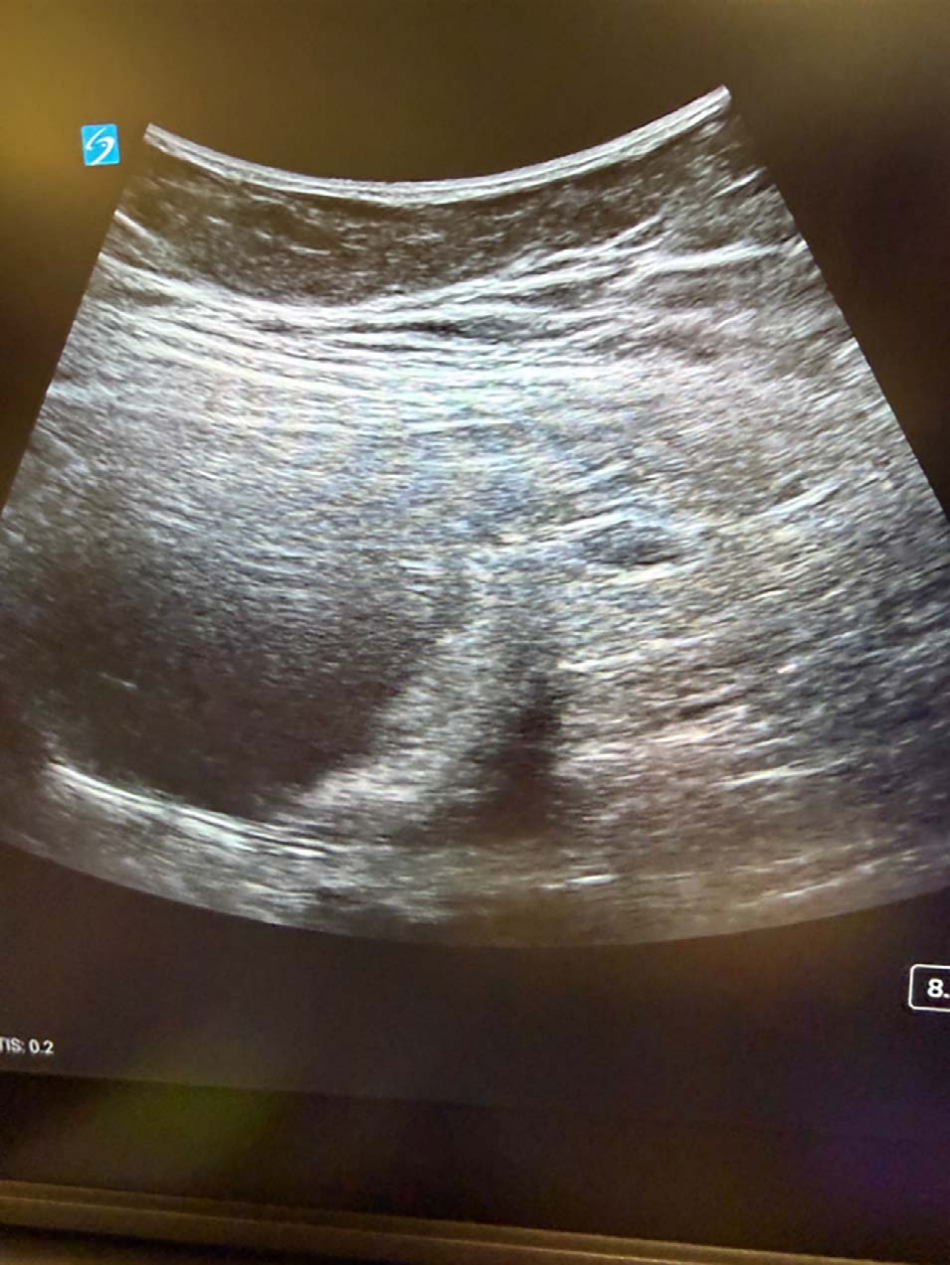
Gastric ultrasound of the stomach with the patient in the supine position. Both hypoechoic and hyperechoic material were identified, a grade 2 finding.

**Figure 2.**
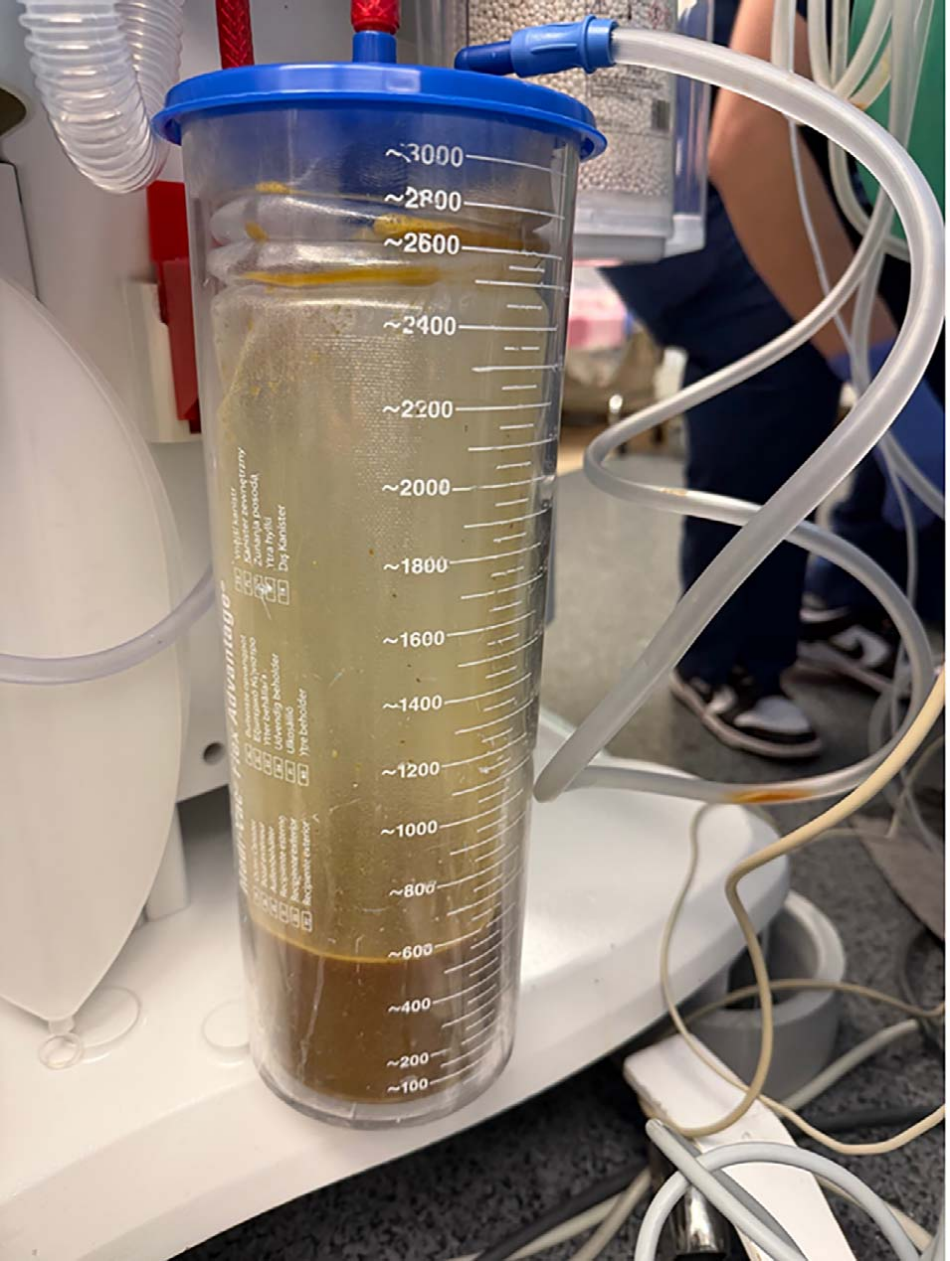
In our patient, 600 mL of gastric contents was successfully decompressed, with an additional similar volume evacuated through the nonsuction port of the nasogastric tube.

Preoperative gastric POCUS of the patient showed substantial gastric contents (grade 2) even after intrahospital fasting for 4 days (Figure 1). Sedation was administered to facilitate nasogastric sump tube placement, resulting in successful decompression of pressurized gastric contents (Figure 2), followed by rapid sequence orotracheal intubation.

The patient underwent exploratory laparotomy with extensive lysis of adhesions, reduction, and primary repair of 2 incarcerated midline ventral hernias (estimated blood loss 20 mL, 1,100 mL crystalloid fluid administration, urine output 240 mL) with no intraoperative vasopressor requirement. Postoperatively, she was extubated and transported to the intensive care unit in an awake, stable state.

Hospital management focused on careful fluid balance and continued nasogastric tube decompression in consideration of the patient's chronic obstructive pulmonary disease, chronic kidney disease, and advanced age. She remained in the hospital for an additional 5 days. Long-term planning included the need for rehabilitation following hospital discharge home.

## DISCUSSION

Gastric POCUS addresses several limitations of traditional methods used to assess aspiration risk by enabling direct, bedside visualization of gastric contents. Using a low-frequency curvilinear probe in the sagittal epigastric plane, the gastric antrum can be identified and characterized based on sonographic appearance, with fluid typically appearing hypoechoic and solid contents appearing more hyperechoic.^4^ This approach allows clinicians to determine whether the stomach is empty or contains clear or solid material and to estimate gastric volumes using validated cross-sectional area measurements.^4-6^

Since its early description in the 2000s, gastric POCUS has evolved into a practical adjunct for perioperative assessment, supported by growing clinical experience and educational recommendations from professional societies.^7,8^ Unlike fasting guidelines that provide population-based estimates and may not reliably reflect individual gastric contents, gastric POCUS provides patient-specific information that can be obtained rapidly at the bedside.^5^

### Diagnostic Performance and Clinical Utility

The diagnostic performance of gastric POCUS has been evaluated across a range of clinical settings. Foundational studies by Perlas et al established that antral cross-sectional area correlates with gastric volume and can be used to differentiate empty from nonempty stomachs.^4^ Subsequent work introduced a qualitative grading system (grade 0 to 2) that provides a simple and reproducible framework for clinical interpretation.^5^

More recent evidence supports the overall accuracy of this approach. A 2024 systematic review and meta-analysis by Pan et al, which included 9 studies and more than 500 patients, demonstrated excellent discriminative ability for identifying nonempty stomachs, with an area under the receiver operating characteristic curve of approximately 0.97 (95% CI 0.95-0.98). The pooled sensitivity was 95% (95% CI 84-99), and specificity was 88% (95% CI 72-95).^9^ These findings indicate that gastric POCUS is highly sensitive for detecting clinically relevant gastric contents, although specificity is lower and may vary depending on patient population and study design.

Importantly, the utility of gastric POCUS extends to patient groups in whom traditional assessment methods are less reliable. In individuals with obesity, for whom physical examination and standard fasting assumptions may be misleading, ultrasound-based estimation of gastric volume has been shown to be feasible and accurate.^10^ Similarly, in patients with conditions associated with delayed gastric emptying, such as diabetes, critical illness, or opioid use, gastric POCUS allows for individualized assessment rather than reliance on fixed fasting intervals.

### Role in Perioperative Decision-Making

**Figure 3.**
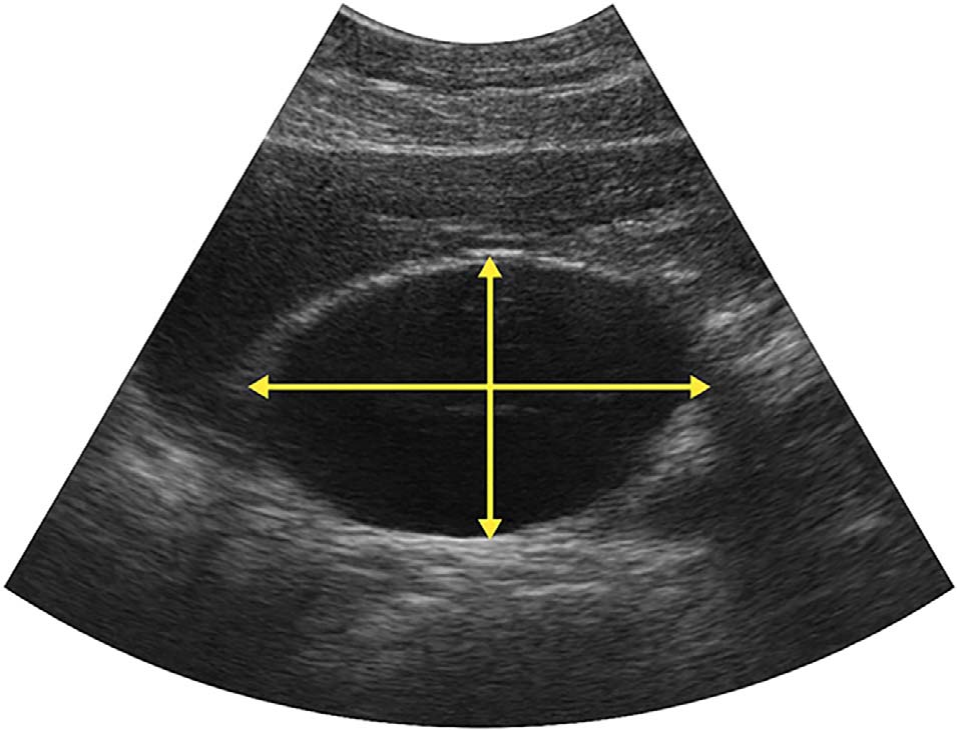
The antral cross-sectional area is depicted on a sagittal point-of-care ultrasound scan of the gastric antrum with the patient in the right lateral decubitus position. The yellow lines are ultrasound measurements of the gastric antrum to allow calculation of intragastric volumes. Gastric volume can be estimated from the right lateral decubitus antral cross-sectional area using the validated Perlas model.^11^ A simple bedside rule is if any antral diameter measurement exceeds 3 cm, the patient is likely to be high risk for pulmonary aspiration, although formal risk assessment should rely upon validated models and clinical context.

A key advantage of gastric POCUS is its ability to provide real-time information that can inform anesthetic management. Identification of large-volume gastric contents or solid material may prompt modification of airway strategy, such as the use of rapid-sequence induction and endotracheal intubation to reduce the risk of pulmonary aspiration. Conversely, confirmation of an empty or low-volume stomach may support proceeding with standard induction techniques, potentially avoiding unnecessary delays or interventions (Figure 3).^11^

Rather than as a replacement for established fasting guidelines, gastric POCUS is best viewed as a complementary tool that refines risk assessment in situations of uncertainty. Gastric POCUS use is particularly relevant in urgent or emergent settings, in patients with unclear fasting histories, and in patients with risk factors for delayed gastric emptying.

### Limitations and Considerations

Despite its advantages, gastric POCUS has limitations. Diagnostic performance is operator-dependent, and appropriate training is required to ensure accurate image acquisition and interpretation.^8^ In addition, most available studies are relatively small and heterogeneous, with variability in patient populations, scanning protocols, and outcome definitions.^9^ While ultrasound can reliably distinguish empty from nonempty stomachs, estimates of gastric volume are approximate and may be less precise at higher volumes or in patients with atypical anatomy.

Furthermore, although gastric POCUS improves identification of patients at potential risk for aspiration, limited direct evidence links the use of gastric POCUS to reductions in aspiration events or perioperative morbidity. As such, clinical decisions should integrate ultrasound findings with the broader clinical context, including patient comorbidities, surgical urgency, and airway considerations.

### Future Directions

Future developments in gastric POCUS are likely to include artificial intelligence (AI)–assisted image analysis to automate identification of the gastric antrum and facilitate real-time estimation of gastric volume.^12,13^ These technologies may improve consistency and reduce operator dependence. In addition, combining ultrasound findings with clinical risk factors may enhance perioperative aspiration risk stratification, supporting more individualized and evidence-informed decision-making.

## CONCLUSION

POCUS for gastric content assessment delivers precise, safe, and efficient care with broad accessibility. Gastric POCUS helps reduce aspiration risk, improves workflow, and is backed by decades of research. Advancements such as AI-assisted cross-sectional area calculations will further strengthen the role of gastric POCUS in perioperative medicine. By improving accuracy, efficiency, and patient safety, gastric POCUS enhances clinical decision-making and makes advanced imaging accessible even in settings with limited resources.

## Notes

*This article meets the Accreditation Council for Graduate Medical Education and the American Board of Medical Specialties Maintenance of Certification competencies for Patient Care and Medical Knowledge.*
